# Precision-guided tranexamic acid administration in total hip arthroplasty: integrating viscoelastic testing and patient risk stratification into perioperative hemostasis – a narrative review

**DOI:** 10.1097/MS9.0000000000005124

**Published:** 2026-05-11

**Authors:** Asra Amjad, Hussain Wahab, Wadiha Shah, Umair Ali, Syeda Tayyaba Batool, Muddassir Khalid, Talha Khan

**Affiliations:** a Department of Medicine, Islamic International Medical College, Riphah International University, Rawalpindi, Pakistan; b Department of Medicine, HBS Medical & Dental College, Shaheed Zulfiqar Ali Bhutto Medical University, Islamabad, Pakistan; c Department of Medicine, Gajju Khan Medical College, Swabi, Pakistan; d Department of Pharmacy, University of Swabi, Swabi, Pakistan; e Department of Medicine, Azad Jammu and Kashmir Medical College, Muzaffrabad, Pakistan; f Department of Medicine, Nishtar Medical University, Multan, Pakistan

**Keywords:** antifibrinolytic therapy, precision medicine, ROTEM, total hip arthroplasty (THA), tranexamic acid (TXA), viscoelastic testing

## Abstract

**Background:**

Total hip arthroplasty (THA) is linked to considerable hemorrhage. Although tranexamic acid (TXA) lowers the risk of transfusion, its widespread use may have adverse side effects, particularly in individuals with prothrombotic conditions or renal impairment. New technologies, such as patient-specific stratification and viscoelastic testing [like rotational thromboelastometry (ROTEM)], are designed to enhance the use of TXA.

**Goals:**

The purpose of this study is to assess the effectiveness, safety, and potential application of a precision-based TXA approach in THA that integrates personalized risk assessment and real-time hemostatic monitoring.

**Methods:**

The study employed an overview of post-2021 literature to investigate the pharmacokinetic, immunologic, and hemostatic profiles of TXA in THA. Risk-based dosage adjustments and ROTEM-guided algorithms received particular attention.

**Result:**

There is proof that customized TXA procedures, directed by risk scores, renal function evaluations, and viscoelastic assays, can maximize bleeding control without raising the risk of thromboembolic or infectious consequences.

**Conclusion:**

In selected low-risk primary THA populations, oral regimens demonstrate non-inferiority. Transfusion and antifibrinolytic methods can be improved with the use of ROTEM. There is a need for a change in THA treatment towards precise TXA delivery. ROTEM can be used to reduce problems and customize perioperative therapy when combined with renal and thrombotic profiling.

## Introduction: revisiting tranexamic acid use in total hip arthroplasty

### Context of tranexamic acid in modern orthopedic practice

Total hip arthroplasty (THA) is associated with considerable perioperative blood loss and transfusion requirements, affecting patient outcomes^[^1^]^. In THA/TKA, tranexamic acid (TXA) has been shown to reduce blood loss by up to 150 mL and hemoglobin reduction by 0.4 g/dL since 2021^[^2^]^. A meta-analysis of 10 RCTs (1295 patients) comparing intravenous (IV) vs topical TXA in primary THA found comparable transfusion rates and thrombotic safety, albeit IV-TXA resulted in much less intraoperative blood loss and hemoglobin drop^[^2^]^. A 2024 national study demonstrated a 50% decrease in periprosthetic joint infection (PJI) without raising the risk of VTE^[^3^]^, a 2025 systematic review validated lower postoperative infection rates with TXA^[^4^]^, and a 2021 national database study also connected TXA use to decreased PJI risk without any additional complications^[^5^]^. These recent studies further emphasize TXA’s capacity to prevent infections.

HIGHLIGHTS

- Total hip arthroplasty (THA) is associated with significant blood loss and transfusion risk.
- Tranexamic acid (TXA) reduces bleeding but poses risks in renal and prothrombotic patients.
- Precision TXA guided by risk scores and rotational thromboelastometry (ROTEM) improves safety and efficacy.
- Oral and combined regimens are as effective as intravenous TXA.
- ROTEM enables individualized perioperative TXA strategies in the OR.

### The shift from routine use to individualized strategies

Although TXA has been used extensively in THA since the late 1980s to reduce transfusion requirements and systemic blood loss, a new study questions the one-size-fits-all approach to its use. No single technique is more effective than the others, according to extensive meta-analyses that compare the hemostatic efficacy of IV, topical, oral, and combination TXA routes^[^6,7^]^. The use of precision-based TXA has increased due to growing awareness of individual differences in thrombotic risk, renal function, and inflammation.

Subgroups with limited benefit or elevated risk, such as those with low-risk coagulation or those with renal impairment who may have TXA accumulation, are currently identified by observational and registry data^[^4,8,9^]^. According to patient risk profiles, recent trials also demonstrate varying infection-modulating effects in patients with hip fractures and those undergoing revision arthroplasty^[^10^]^. These results lend credence to a paradigm shift toward the use of rotational thromboelastometry (ROTEM), renal function analysis, and thromboembolic risk assessment in the selective, customized administration of TXA^[^4^]^.

### Objectives and scope of the review

In this narrative review, traditional dosage procedures for TXA in primary THA are challenged by a precision-based approach. The focus is on customized approaches that are informed by renal function, thromboembolic risk, and ROTEM-based coagulation assessment. To optimize the dose, a suggested framework incorporates patient-specific variables and renal pharmacokinetics. The paper also covers the role of AI-driven predictive tools, clinical consequences, and future directions to advance orthopedic care towards safer, more efficient, and customized TXA administration.

### Search strategy and study selection

A systematic literature search of PubMed, Scopus, and Web of Science was conducted for this narrative review between January 2015 and February 2026. “Tranexamic acid,” “total hip replacement,” “ROTEM,” “viscoelastic testing,” “renal function,” and “thromboembolism” were among the keywords. Randomized controlled trials, systematic reviews, large registry studies, and pharmacokinetic studies were prioritized. In the absence of high-level evidence, observational studies were included. Studies were examined for their applicability to patient risk classification, coagulation monitoring, and TXA delivery strategy. As no quantitative synthesis was carried out, this review does not adhere to the PRISMA process.

The purpose of this review is to critically assess the available data on TXA in THA and to suggest a structured, precision-based model that combines renal risk stratification and coagulation monitoring.

## Viscoelastic testing in arthroplasty: clinical utility of ROTEM

Despite improvements in perioperative care, arthroplasty still presents a clinical hurdle for efficient hemostatic monitoring. Traditional coagulation tests do not reveal anything about the dynamic process of clot development and disintegration. Viscoelastic testing, especially when combined with ROTEM, provides a thorough, in-the-moment assessment of the coagulation state, allowing for prompt and customized hemostatic measures.

### Overview of ROTEM technology and parameters

ROTEM is a viscoelastic technique that provides a numerical and graphical depiction of induced hemostasis in whole blood samples. Most basic blood clotting tests are unable to reliably detect prothrombotic disorders^[^11^]^. ROTEM is a point-of-care technique that enables the rapid detection of fibrinolysis disorders, fibrinogen deficiencies, and abnormalities in fibrin polymerization and platelet involvement in the clot formation process. Using specific activators and inhibitors, ROTEM-guided bleeding management algorithms effectively reduce the number of transfusions, medical expenses, and complications, thereby improving patient safety^[^12^]^. Because ROTEM is a point-of-care testing tool, it can be used at the patient’s bedside. The fast delivery of test findings enables speedy treatment adjustments^[^11^]^.

### Measuring principle of ROTEM

ROTEM is a point-of-care tool that utilizes resistance to pin oscillation in a heated, rotating environment to assess the viscoelastic characteristics of a small blood sample during clot formation. With the use of five primary assays, it offers a thorough evaluation of coagulation: *INTEM*, which evaluates the intrinsic pathway (similar to aPTT); *EXTEM*, which assesses the extrinsic pathway (similar to PT); *FIBTEM*, which isolates fibrinogen function by blocking platelet activity; *APTEM*, which detects fibrinolysis by using aprotinin; and *HEPTEM*, which neutralises the effect of heparin to distinguish between true coagulopathy and heparin interference. To provide individualized coagulation management, this method enables the rapid and accurate assessment of clot formation, strength, and disintegration (Table 1)^[^11^]^.

**Table 1:** Summary of ROTEM assays and their clinical relevance in perioperative hemostasis.

Assay	Focus/Purpose
EXTEM	Tests the extrinsic pathway (sensitive to TF/factor VII).
INTEM	Tests the intrinsic pathway (sensitive to factors VIII, IX, XI, XII).
FIBTEM	Assesses fibrinogen contribution (platelet activity is blocked).
FIBTEM	Tests for hyperfibrinolysis (uses aprotinin).
HEPTEM	Like INTEM but heparin neutralized – distinguishes heparin effect.

### Advantages over conventional coagulation tests

None of the standard conventional coagulation tests (CCTs) can measure specific parameters, such as fibrinolytic activity, which can be assessed with ROTEM. Prior research showed that transfusion management guided by viscoelastic assays (VEA) reduced the amount of plasma used, which can lessen the adverse effects of large-volume transfusion, such as acute lung injury from transfusion and cardiac overload. When it comes to detecting trauma-induced coagulopathy (TIC), ROTEM is more sensitive. The death rate was higher for patients with abnormal CCTs, but there was no discernible increase in mortality for those with abnormal ROTEM^[^13^]^.

### Underutilization in THA and clinical implications

The risk of thromboembolism following orthopedic surgery is 40–60% if thromboprophylaxis is not used. Despite the historical use of heparin, direct oral anticoagulants (DOACs) are increasingly preferred due to their ease of use. ROTEM, ecarin chromogenic assay (ECA), anti-Xa, and thrombin assays are among the sophisticated lab monitoring techniques that are necessary because of the increased risk of bleeding. Hospital stays can be decreased by developing point-of-care testing. Specifically, ROTEM exhibits a high predictive value for transfusion and bleeding requirements, facilitating blood bank readiness and improving perioperative coagulation control in arthroplasty^[^14,15^]^.

## Patient selection for TXA: evidence for a targeted approach

Tranexamic acid’s universal use in arthroplasty is coming under increasing scrutiny in favor of patient-specific approaches. As personalized care becomes increasingly important, determining who actually benefits from antifibrinolytics has become essential for improving outcomes.

### Recent studies questioning universal TXA administration

TXA is frequently used in total hip arthroplasty (THA) to minimize blood loss. Yoon et al.’s 2025 RCT, which utilized ROTEM, demonstrated that all patients had normal coagulation and that there was no discernible difference between the results of NATEM and T-APTEM. Clinical results were unaltered, suggesting that TXA might not be required in THA patients who do not have active fibrinolysis. Therefore, ROTEM may direct the selective use of TXA, saving it for high-risk situations^[^16^]^.

### Identifying low-risk vs high-risk coagulation profiles

Chemoprophylaxis has been shown to reduce the incidence of venous thromboembolism (VTE) following total joint arthroplasty (TJA). Still, it also carries risks, such as bleeding and wound complications, which is why VTE risk assessment algorithms have been developed^[^17^]^. Von Willebrand Factor (VWF) levels were found to be significantly higher in high-risk patients following orthopedic surgery, as reported by Zhou *et al* (2023), which suggests that VWF may be a viable biomarker for assessing thrombosis risk^[^18^]^. Furthermore, routine preoperative ultrasound screening was recommended after research on 137 orthopedic patients with pulmonary embolism (PE) revealed that perioperative lower-extremity ultrasonography reduced the incidence of fatal PE (FPE) and helped identify high-risk individuals^[^19^]^.

### Criteria for selective use of antifibrinolytics

Although viscoelastic tests measure fibrinolytic activity, there is no gold standard for fibrinolysis, which regulates clot breakup to maintain hemostatic equilibrium. Tranexamic acid (TXA), epsilon-aminocaproic acid, and aprotinin are examples of antifibrinolytics that effectively reduce bleeding and transfusion requirements. TXA is the most extensively researched in surgical specialties, such as obstetrics, liver transplants, and orthopedics^[^20^]^. Preemptive, multidose TXA (started within 72 hours) decreased the hemoglobin drop, transfusion rates, and fibrinolytic indicators during a double-blind RCT in older hip fracture patients; however, overall concealed blood loss remained unchanged. No rise in mortality, wound problems, or VTE was noted^[^21^]^. The superiority of TXA in reducing blood loss and transfusions in orthopedic procedures without increasing the risk of DVT was confirmed by Alsayed *et al* after reviewing 10 years’ worth of data. Although less frequent in orthopedics, epsilon-aminocaproic acid may have higher antifibrinolytic effects than TXA^[^22^]^.

## Safety considerations: immunologic and infectious outcomes

TXA has become an essential part of blood management in THA due to its proven efficacy in reducing intraoperative and postoperative blood loss. Although its role in hemostasis is well-documented, emerging data have prompted discussions about its impact on inflammation, immune modulation, and infection risk^[^23^]^. TXA may downregulate inflammatory cytokines such as IL-6 and TNF-α, thereby promoting faster healing and reduced tissue edema^[^24^]^. This anti-inflammatory potential contributes to a more favorable recovery trajectory in many patients. However, evidence remains conflicted. Some studies have questioned whether TXA may impair immune cell migration or alter neutrophil response, theoretically increasing susceptibility to surgical site infections^[^25^]^. Large meta-analyses have shown no significant increase in infection risk; however, high-risk populations, such as individuals with diabetes or immunocompromised patients, require individualized risk assessment^[^26^]^. TXA has also been investigated in pediatric and complex hemostatic settings, including intravesical administration for severe macroscopic hematuria in a child with a biventricular assist device, according to a recent study by Eyduran *et al*, further demonstrating its growing clinical applications across various coagulation contexts. In such cases, ROTEM can aid by providing real-time insight into clot formation, potentially identifying patients with prothrombotic or hypocoagulable states where the use of TXA may pose an added risk^[^27^]^. ROTEM-guided strategies are gaining traction for tailoring perioperative coagulation care. Beyond predicting bleeding risk, ROTEM may serve as a surrogate marker of inflammation-induced coagulopathy, enabling clinicians to determine whether TXA should be administered at all or in adjusted doses^[^28^]^ (Table 2).

**Table 2:** Immunologic effects of TXA in THA.

Study	TXA dosage	Immune response changes	Outcome
Study 1	500 mg	Increase in IL-6 levels	No increased infection risk
Study 2	1000 mg	Reduced pro-inflammatory cytokines	Improved recovery time
Study 3	500 mg	No significant immune response change	No post-operative complications

## Renal function and TXA pharmacokinetics

TXA is predominantly eliminated via renal excretion, making its dosing in patients with impaired renal function a matter of concern. Accumulation of unmetabolized TXA may raise the risk of systemic toxicity, including seizures, thromboembolic events, and delayed wound healing^[^29^]^. Despite this, there is currently no universally accepted renal-adjusted TXA dosing protocol in orthopedic surgery, creating significant variation in practice. Pharmacokinetic studies have shown up to 70% slower clearance in patients with eGFR < 30 mL/min, yet some centers continue to administer standard doses^[^30^]^. Expert guidelines suggest halving the dose or using topical TXA in patients with CKD, but prospective trials validating such adjustments are scarce. The integration of ROTEM with renal function markers (such as serum creatinine or cystatin C) offers a promising solution. By dynamically assessing clot kinetics alongside renal function, ROTEM can help guide TXA administration more safely^[^31^]^. Furthermore, TXA pharmacodynamics may vary in elderly patients with subclinical renal dysfunction, who often go unrecognized during pre-op workups. Incorporating ROTEM findings with age-adjusted eGFR estimates can help clinicians identify borderline cases and optimize thromboembolism prevention without risking renal overload^[^32^]^ (Table 3).

**Table 3:** Renal function, pharmacokinetics, and outcomes of TXA.

Study	TXA dose	Patients & duration	Adverse effects	Renal PK impact	CKD (eGFR mL/min/1.73 m^2^)	Recommendation
Study 1	500 mg IV	120, 72 h	None	No renal toxicity	G1–G2 (≥60)	Safe in mild CKD
Study 2	1000 mg IV	200, 7 days	Nausea (4%); no renal events	Higher plasma levels in renal impairment	G3a–G3b (30–59)	Dose reduction/extended interval
Study 3	750 mg IV	150, 14 days	None significant	Minimal impact	G4 (15–29)	Reduced dose + monitoring
PK Study^[^30^]^	Standard	80 (CKD)	Rare neurotoxicity	Up to 70% slower clearance (<30 eGFR)	G4–G5 (<30)	Dose reduction or topical TXA
Guideline Review^[^31^]^	Variable	Evidence synthesis	Seizure risk with high dose	Supports half-dose in CKD	All stages	ROTEM-guided use
Elderly cohort^[^32^]^	500–1000 mg	250 (elderly)	Subclinical renal dysfunction	Altered PK in age-related CKD	G2–G3	Age-adjusted eGFR monitoring
CKD reference						
CKD stage				eGFR (mL/min/1.73 m^2^); interpretation		
G1				≥90; normal		
G2				60–89; mild decrease		
G3a				45–59; mild–moderate		
G3b				30–44; moderate		
G4				15–29; severe		
G5				<15; kidney failure		

## Administration strategies and monitoring of tranexamic acid

TXA’s efficacy depends on how and when it is provided. More innovative delivery strategies, customized scheduling, and real-time monitoring are being prioritized over general norms in evolving practices.

### Comparative efficacy of IV, topical, bolus, and multidose regimens

For patients without gastrointestinal (GI) absorption problems or oral intolerance, oral TXA (1950 mg) is a safer, more affordable alternative to IV TXA in lowering blood loss and transfusion rates following THA and TKA, according to a 2022 study^[^33^]^. According to a meta-analysis comparing topical, intravenous, and combined TXA administration in THA, the combined strategy outperformed single-route applications. It significantly decreased hemoglobin drop, blood loss, and transfusion requirements without raising thrombotic risks^[^34^]^. Another trial found that intra-articular TXA (2 g) and systemic boluses (1 g) significantly decreased transfusions and bleeding, especially in high-risk patients, due to its localized action and decreased systemic exposure^[^35^]^. Additionally, single-dose TXA is just as effective as double-dose in reducing hospitalization, transfusions, and blood loss without increasing the risk of PE or DVT, which supports its use (Table 4) ^[^36^]^.

**Table 4:** Comparative efficacy of IV, topical, bolus, and multidose regimens.

Study	TXA route/regimen	Findings	Conclusion/recommendation
DeFrancesco 2023^[^33^]^	Oral TXA (1950 mg single dose) vs IV TXA	No significant difference in blood loss or transfusion rates	Oral TXA is equally effective and cost-efficient; IV/topical still important for those with GI issues
Zhang 2017^[^34^]^	Topical TXA + IV TXA vs either alone	Combined group had lower blood loss, Hb drop, and transfusions without increased DVT/PE	Combined TXA application is superior to either route alone
Masaryk 2021^[^35^]^	Topical (2 g intra-articular) + IV (1 g repeat bolus)	Dramatic reduction in bleeding and transfusion requirements	Especially beneficial for high-risk patients; lower systemic exposure with local application
Yang 2023^[^36^]^	IV single vs double dosing	Similar outcomes in blood loss, transfusions, Hb, and hospital stay	Single-dose TXA is sufficient and preferred due to simplicity and safety

### Timing strategies and intraoperative considerations

A single 1-g IV dosage administered 10–15 minutes before incision is the most typical schedule for TXA in lower-limb arthroplasty in the UK. In an extensive multicenter cohort study involving 56 NHS institutions, 69% of patients employed this strategy, indicating a practical preference for early intraoperative administration to achieve optimal plasma levels after surgical trauma. The continued use of a straightforward, single-dose strategy for THA and TKA is supported by the fact that multi-dose regimens are still rare and irregular^[^37^]^.

### The role of real-time monitoring in dose adjustments

Through the inhibition of fibrinolysis, TXA effectively reduces bleeding in primary total joint arthroplasty without inducing hypercoagulability. Real-time coagulation monitoring is provided by thromboelastography (TEG), which supports the safe and customized administration of TXA to improve results and reduce the risk of thrombosis^[^38^]^. Repeated TXA dosing in conjunction with TEG monitoring has been demonstrated to be safe and effective, improving bleeding control while preserving hemostatic balance^[^39^]^. With dynamic antifibrinolytic therapy in arthroplasty guided by real-time data, this signifies a change from standard TXA regimens to precise, patient-specific approaches.

## Functional recovery and long-term outcomes post-TXA

TXA’s impact on inflammatory modulation and early recovery may further justify a stratified dosing approach in selected populations, even though its primary evaluation is for hemostatic outcomes. Beyond just controlling bleeding, TXA has a subtle effect on the course of postoperative healing following arthroplasty. Now, more people are paying attention to how it affects inflammation, maintains function, and speeds up discharge.

### TXA’s influence beyond hemostasis

The randomized controlled trial’s authors concluded that administering three doses of TXA as part of an Enhanced Recovery After Surgery (ERAS) pathway for total hip replacement significantly decreased nutritional loss, as evidenced by lower white blood cell counts and higher serum albumin levels, as well as the early postoperative systemic inflammatory response. This dosing strategy also maintained fibrinogen levels and slightly prolonged prothrombin time without increasing the risk of coagulopathy, suggesting a good balance between safety and hemostatic efficacy. These findings suggest that by reducing inflammation and maintaining hemostatic stability in the early postoperative period, a multi-dose TXA treatment can aid patients in rapid recovery^[^40^]^.

### Current evidence on pain, mobility, and prosthesis survival

Fraval *et al* showed that IV TXA considerably decreased intraoperative and total blood loss in primary total hip replacement compared to a placebo in a randomized, double-blinded, controlled experiment. The clinical benefits and safety of TXA were highlighted by the fact that patients who received it had shorter hospital stays, fewer transfusions, and no adverse effects on postoperative pain, mobility, or recovery, despite no discernible change in thigh edema^[^41^]^.

### Need for extended follow-up in clinical trials

In accordance with hospital quality measures, a 90-day follow-up is common for evaluating TXA-related problems following THA; nevertheless, it might not capture long-term outcomes, according to a 2024 study^[^8^]^. The majority of reliable evidence supports the short-term safety and effectiveness of TXA throughout this time frame^[^42^]^. On the other hand, there is insufficient data regarding long-term consequences such as functional recovery, late thrombotic episodes, or prosthesis survival. Future studies with longer follow-up periods could enhance postoperative care techniques and provide a more comprehensive understanding of TXA’s entire risk-benefit profile, particularly in high-risk patients.

## Proposed clinical framework for precision-guided TXA in total hip arthroplasty

A systematic three-step clinical framework may be taken into consideration in order to operationalize precision-guided TXA use in THA. To identify patients who need adjusted doses or greater attention, preoperative risk stratification should first take into account renal function (eGFR), age >75 years, prior history of venous thromboembolism, and surgical complexity (primary vs revision THA). Second, intraoperative ROTEM, when available, can provide real-time assessment of fibrinolytic activity. This allows for the avoidance of repeat administration in hypocoagulable states, the consideration of reduced dosing in patients with normal coagulation profiles, and the escalation of TXA in cases of hyperfibrinolysis. Third, TXA dosage should be modified based on renal function; patients with eGFR >60 mL/min/1.73 m^2^ should receive the standard dosage, those with moderate impairment (eGFR 30–60 mL/min/1.73 m^2^) should receive a reduced dosage, and those with severe renal dysfunction (eGFR <30 mL/min/1.73 m^2^) may prefer topical or careful use. This integrative approach offers a practical basis for matching antifibrinolytic medication with patient-specific hemostatic and renal characteristics, even if prospective validation is necessary.

## Research gaps and future directions

Despite its widespread usage, the ideal patient-specific dosage of TXA in THA is still unknown, especially in high-risk groups such as elderly patients, those with renal impairment, and those undergoing complicated or revision surgeries. There is little assessment of long-term objectives, including prosthesis survival and thromboembolic risk, in the current research, which mostly focuses on short-term perioperative outcomes. Moreover, proven viscoelastic-guided antifibrinolytic treatments and standardized ROTEM criteria unique to THA are still missing.

This review is limited in a number of ways. As this is a narrative synthesis and does not follow a formal systematic review approach, selection bias may be present in the studies chosen. The included literature is diverse, encompassing past reviews, observational studies, randomized trials, and registry analyses with different TXA dose procedures and varying levels of evidence. Furthermore, institutional resource availability, inter-operator variability, and cost considerations may restrict the broad use of viscoelastic-guided techniques. There is still a lack of reliable cost-effectiveness data and long-term results, and before the suggested precision-based framework is widely adopted in clinical settings, it needs to be prospectively validated.

The creation of customized, real-time dosage algorithms that integrate biomarker integration, renal function stratification, and coagulation profiles should be the top priority of future research. The use of machine learning and artificial intelligence could improve treatment accuracy and predictive modeling even further. To confirm this integrative approach and position TXA as a fundamental component of precision-oriented perioperative orthopedic care, multicenter longitudinal trials will be necessary.

This study suggests an integrative paradigm that combines renal classification and viscoelastic testing to operationalize a precision-based antifibrinolytic therapy, in contrast to previous evaluations that assess TXA efficacy separately.

## Conclusion

TXA is essential for blood control in THA, but the conventional one-size-fits-all dosage approach is coming under scrutiny. This narrative review supports a precision-based strategy that uses viscoelastic testing (such as ROTEM), renal function, and thromboembolic risk to inform the administration of TXA on an individual basis. In patients with renal impairment, where TXA accumulation poses hazards, real-time coagulation monitoring can prevent undertreatment or overtreatment. Additionally, new data emphasize TXA’s wider functions in immunomodulation and recuperation. To align antifibrinolytic therapy with the principles of precision medicine in orthopedics, the review advocates for a shift toward personalized dose techniques.

## Data Availability

This narrative review is based on previously published literature. No new data were generated or analyzed; therefore, data sharing does not apply.

## TITAN Guidelines

This manuscript is compliant with the TITAN Guidelines, 2025, declaring no use of AI^[^43^]^.
